# Intensity-Modulated Proton Therapy for an Unresectable Giant Non-functioning Pituitary Adenoma: A Case Report and Literature Review

**DOI:** 10.7759/cureus.92987

**Published:** 2025-09-23

**Authors:** Bo Yan, Shuihua Wu, Jiwei Bai, Shikai Wu, Xijia Zhang, Yue Zou, Dongxue Zhou, Jie Wang, Zisheng Wang, Wei Wang, Zhenmin Fu, Lu Yang, Masashi Mizumoto, Hideyuki Sakurai, Shosei (Xiangxing) Shimizu (Qingshui)

**Affiliations:** 1 Department of Otorhinolaryngology - Head and Neck Surgery, Xuanwu Hospital, Capital Medical University, Beijing, CHN; 2 Department of Neurosurgery, Hunan Children's Hospital, Changsha, CHN; 3 Department of Neurosurgery, Beijing Tiantan Hospital, Capital Medical University, Beijing, CHN; 4 Department of Medical Oncology, Peking University First Hospital, Beijing, CHN; 5 Department of Pediatric Radiation Therapy Center, Hebei Yizhou Cancer Hospital, Zhuozhou, CHN; 6 Department of Radiotherapy Physics and Technology, Hebei Yizhou Cancer Hospital, Zhuozhou, CHN; 7 Department of Radiology, Hebei Yizhou Cancer Hospital, Zhuozhou, CHN; 8 Department of Radiation Oncology, University of Tsukuba Hospital, Tsukuba, JPN; 9 Department of Proton Beam Therapy Center, University of Tsukuba Hospital, Tsukuba, JPN

**Keywords:** giant adenoma, intensity-modulated proton therapy (impt), non-functioning pituitary adenoma (nfpa), proton beam therapy (pbt), radiotherapy

## Abstract

Giant non-functioning pituitary adenomas (NFPAs) often extend into or compress critical structures, such as the optic nerves, brainstem, and cavernous sinus, frequently making complete surgical resection difficult or unfeasible and posing significant therapeutic challenges. Radiotherapy (RT) is crucial for unresectable or residual disease, yet conventional techniques may struggle to deliver adequate doses while sparing organs at risk (OARs). Proton beam therapy (PBT), particularly intensity-modulated proton therapy (IMPT), offers potential dosimetric advantages, such as superior conformity and OAR sparing. This report presents an IMPT case for a giant, recurrent, unresectable NFPA and reviews the PBT literature, focusing on its advantages and role in NFPA management.

A 49-year-old female presented with headache and progressive visual decline, approximately six years after her second surgery for a pituitary adenoma, which was first diagnosed in 2013. Contrast-enhanced magnetic resonance imaging (MRI) revealed a giant (>5 cm), unresectable, recurrent NFPA extending into the cavernous sinus and compressing the optic chiasm, brainstem, and right temporal lobe, and encasing the optic nerves and internal carotid arteries. Given the tumor's complex geometry and the patient's age, IMPT (54 Gy RBE (relative biological effectiveness)/30 fractions) was administered. MRI performed approximately 16 months post-IMPT showed significant tumor reduction and relief of brainstem and optic chiasm compression, indicating effective local control without acute high-grade toxicity.

The physical properties of protons, characterized by the Bragg peak, allow for superior dose conformity and sparing of critical OARs. In this complex case, IMPT demonstrated excellent tumor control. This reduction in integral dose to healthy tissue theoretically lowers long-term risks, particularly the development of secondary malignant neoplasms (SMNs), making IMPT an advantageous modality for challenging NFPAs - especially in younger patients with long life expectancies. Further prospective studies are needed to clinically validate these long-term benefits and solidify IMPT's role in the treatment of pituitary adenomas.

## Introduction

Pituitary adenomas, benign monoclonal neoplasms arising from adenohypophyseal cells, account for 10%-20% of primary intracranial tumors [1,2]. Non-functioning pituitary adenomas (NFPAs), which lack hormone hypersecretion syndromes, represent a substantial subgroup, comprising between 14% and 54% of pituitary adenomas [1,3,4]. Their insidious onset often leads to diagnosis at larger sizes (macroadenomas ≥10 mm; giant adenomas >40 mm) [3,5], frequently exhibiting extension into critical structures and making complete surgical removal difficult [1,6,7]. Giant pituitary adenomas, conventionally defined as tumors exceeding 4 cm in maximum diameter [5], commonly exhibit extension and compressive behavior. Complete surgical resection is particularly difficult in cases with extension into critical structures such as the optic nerve, cavernous sinus, and brainstem, thereby complicating attempts at total removal [8,9].

NFPAs typically manifest through mass effect on adjacent structures [1,10]. Common presentations include visual field deficits (often bitemporal hemianopia) from optic pathway compression (reported in 40%-70% of patients), non-specific headaches (16%-75%), and hypopituitarism due to pituitary compression (affecting 37%-85% of patients with macroadenomas) [1,3]. Diagnosis integrates clinical findings, endocrine assessment, ophthalmological evaluation (including perimetry), and high-resolution magnetic resonance imaging (MRI) [10,11].

Transsphenoidal surgery (TSS) is the primary treatment for symptomatic NFPAs, aiming for neural decompression and tissue diagnosis [11,12]. However, complete resection is often challenging for large tumors or those causing significant compression or extending into critical areas, leading to frequent residual or recurrent disease (recurrence rates post-surgery ranging from 12% to 66%) and necessitating effective adjuvant therapies [11,13,14].

Radiotherapy (RT) is critical for managing residual, recurrent, or unresectable NFPAs [7,15]. Techniques have evolved from conventional fractionation radiotherapy (CFRT) to highly conformal photon methods such as stereotactic radiosurgery (SRS), fractionated stereotactic radiotherapy (FSRT), intensity-modulated radiation therapy (IMRT), and volumetric modulated arc therapy (VMAT) [10,16]. More recently, proton beam therapy (PBT) has emerged as an alternative, utilizing the Bragg peak to concentrate dose at the target while minimizing exit dose, thus sparing distal normal tissues [17,18]. This physical advantage is particularly beneficial near radiosensitive structures like the optic nerves and brainstem [19,20]. Intensity-modulated proton therapy (IMPT), the most advanced PBT technique using pencil beam scanning (PBS), offers superior conformity via sophisticated dose optimization, especially advantageous for complex geometries near critical structures, enhancing normal tissue protection [17,21].

In this report, we present a case of a giant, recurrent NFPA, deemed unresectable due to extension into and compression of the optic chiasm, brainstem, and cavernous sinus, following two prior surgical resections. This complex case was successfully managed using IMPT, demonstrating both safety and efficacy. Furthermore, we provide a comprehensive review of the pertinent literature, comparing IMPT and other RT modalities applicable to NFPAs, with specific attention to the distinctions between passive scattering and intensity-modulated proton techniques, and the comparative risks of long-term toxicities, particularly secondary malignant neoplasms (SMNs).

## Case presentation

**Figure 1:**
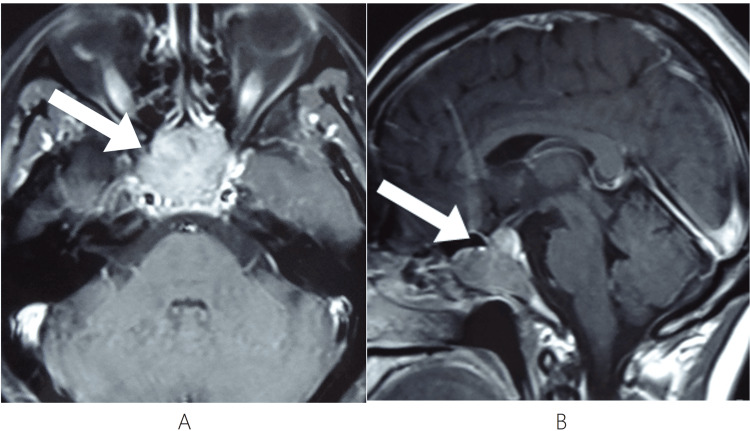
Pre-surgery Baseline - March 2014 A mass with an abnormal signal is seen in the sellar region, compressing the optic chiasm superiorly. (A) Axial T1-weighted post-contrast image shows a sellar mass with heterogeneous mild-to-moderate enhancement (arrow). (B) Sagittal T1-weighted post-contrast image shows the lesion with heterogeneous mild-to-moderate enhancement (arrow).

A 49-year-old female presented with headache and worsening vision approximately six years after her second surgery for a pituitary adenoma. Her history began with an incidental sellar lesion finding in October 2013. An MRI in March 2014 (pre-surgery baseline) showed a sellar mass measuring approximately 3.7 cm × 3.9 cm × 2.8 cm, with heterogeneous mild-to-moderate enhancement, compressing the optic chiasm superiorly (Figure 1).

**Figure 2:**
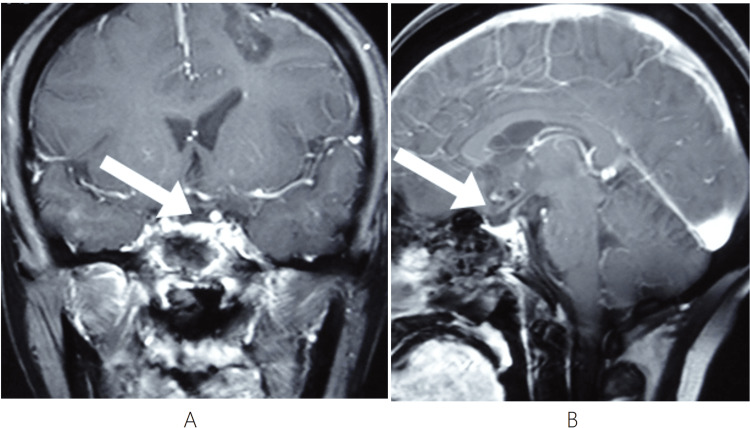
Post-surgery - March 2014 Definite residual disease in the sellar region post-surgery. (A) Coronal T1-weighted post-contrast image shows linear enhancement in the post-surgical sellar area (arrow). (B) Sagittal T1-weighted post-contrast image shows linear enhancement in the post-surgical sellar area (arrow).

Five months after the initial finding (March 2014), she underwent endoscopic endonasal resection for worsening pain; postoperative pathology confirmed a pituitary adenoma. Immunohistochemistry (IHC) revealed LH(-), FSH(-), ACTH(+), PRL(±), TSH(-), GH(-), with a Ki-67 proliferation index of 2%+. A post-surgery MRI in March 2014 showed linear enhancement in the post-surgical sellar area, with definite residual disease (Figure 2). Following a multidisciplinary discussion, a strategy of active surveillance was adopted, with adjuvant RT reserved for evidence of tumor progression.

Approximately 41 months post-first surgery (August 2017), progressive visual decline prompted investigation, revealing tumor recurrence. A second endoscopic resection targeting the recurrent tumor in the right cavernous sinus and sellar region was performed one month later (September 2017). Pathology again confirmed pituitary adenoma; IHC results were LH(-), FSH(+), ACTH(-), PRL(-), TSH(-), GH(-), Collagen IV(-), CK18(+), P53(-), with a Ki-67 index of 5%+. The elevated Ki-67 index (≥3%) in this second sample suggested potentially higher proliferative activity and an increased risk of recurrence [22]. Given the patient's stable clinical condition postoperatively, a continued strategy of active surveillance was recommended.

**Figure 3:**
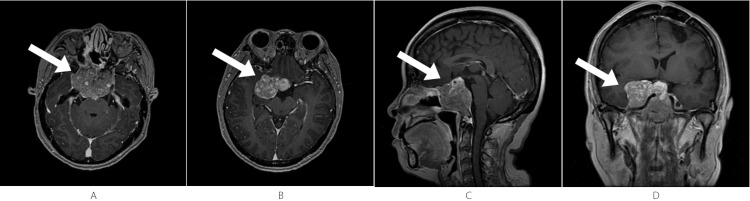
Pre-IMPT - October 2023 The lesion involves the clivus and bilateral cavernous sinuses, encasing both internal carotid arteries. The lesion compresses the brainstem, and the optic chiasm is compressed and displaced superiorly. Both optic nerves are encased by the lesion. (A, B) Axial T1-weighted post-contrast images show a sellar mass with heterogeneous enhancement (arrow). (C): Sagittal T1-weighted post-contrast image shows the lesion with heterogeneous mild-to-moderate enhancement (arrow). (D) Coronal T1-weighted post-contrast image shows the lesion with heterogeneous mild-to-moderate enhancement (arrow). IMPT, intensity-modulated proton therapy

After several years of stability, an MRI performed approximately six years after the second surgery (October 2023, pre-IMPT) confirmed significant tumor recurrence/progression (Figure 3). The MRI revealed a giant (>5 cm) sellar mass measuring approximately 5.7 cm × 3.7 cm × 4.6 cm, showing heterogeneous mild-to-moderate enhancement (Figures 3A, 3B). The tumor demonstrated extensive extension: superiorly, it involved the optic chiasm (which was compressed and displaced superiorly) and bilaterally encased the optic nerves; laterally, it extended into the bilateral cavernous sinuses, encasing both internal carotid arteries; posteriorly, it extended to involve the clivus and compressed the brainstem; additionally, it compressed the right temporal lobe, rendering it unresectable (Figures 3C, 3D). 

**Figure 4:**
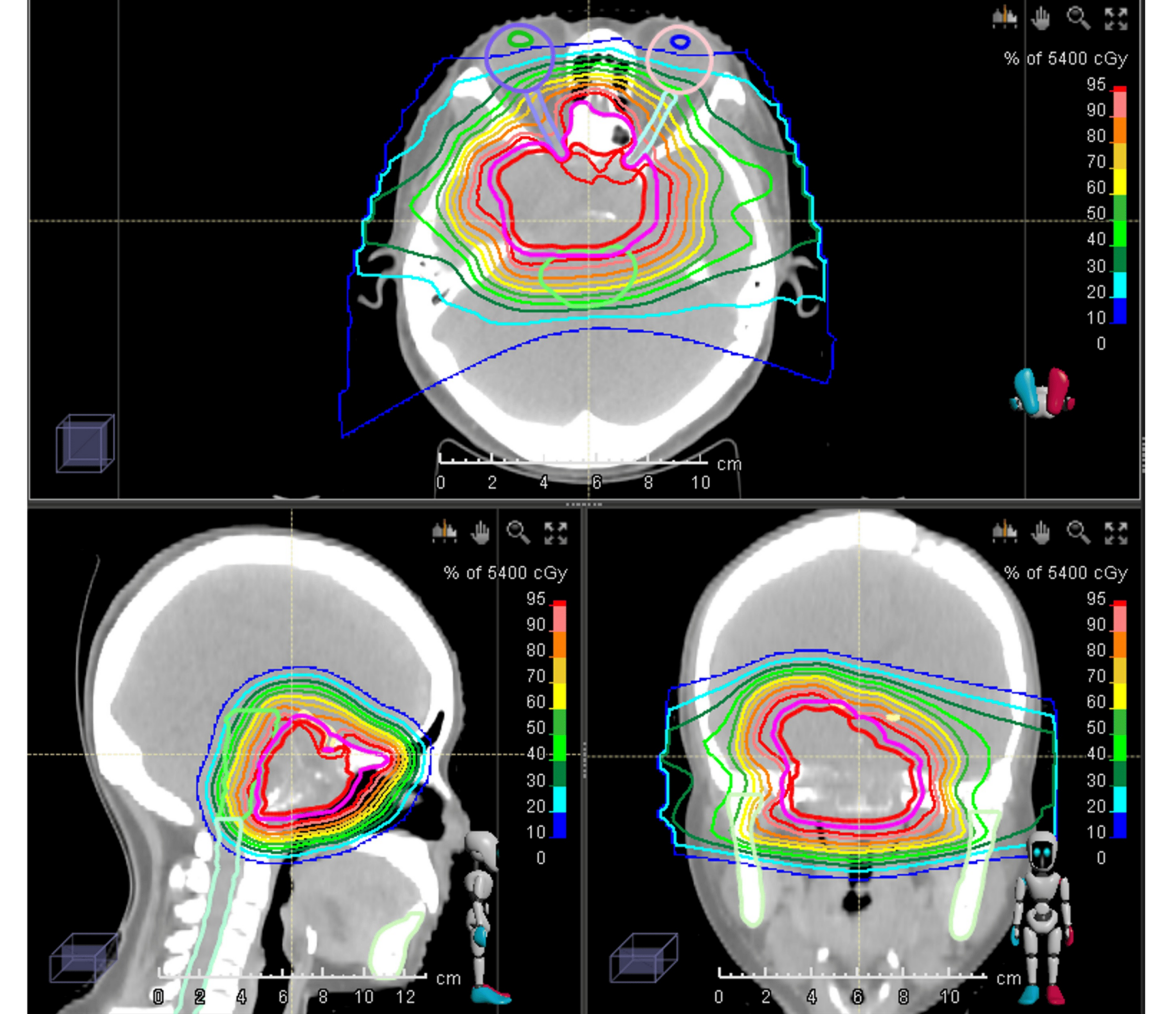
Target Volume Delineation and Planning Image Fusion: Planning CT and planning MRI scans were co-registered for target definition. GTV: The solid tumor, located in the sellar region, was delineated as the GTV based on fused imaging. CTV: The CTV was generated by adding a 2 mm isotropic margin around the GTV. Treatment Plan Prescription: The prescribed dose to the CTV was 54 Gy in 30 fractions (CTV: 54 Gy/30 f). Dose Constraint: The maximum dose to the optic chiasm was constrained to <52 Gy. GTV, gross tumor volume; CTV, clinical target volume; CT, computed tomography; MRI, magnetic resonance imaging

An endocrine evaluation upon admission revealed no clinically significant abnormalities in pituitary hormone levels, consistent with the diagnosis of a non-functioning adenoma. A formal ophthalmology consultation during admission documented specific visual acuity of 0.5 in the right eye and 0.8 in the left eye. Perimetry testing confirmed the absence of hemianopia. Given the recurrence, size, compressive effects, and surgical risks, the patient was referred for RT. IMPT was selected following multidisciplinary consultation. The gross tumor volume (GTV) encompassed the solid tumor, and a clinical target volume (CTV) was generated with a 2 mm isotropic margin. She received 54 Gy (relative biological effectiveness, or RBE), prescribed to the CTV in 30 fractions over six weeks (October-November 2023), with the optic chiasm dose constrained to <52 Gy (Figure 4). 

**Figure 5:**
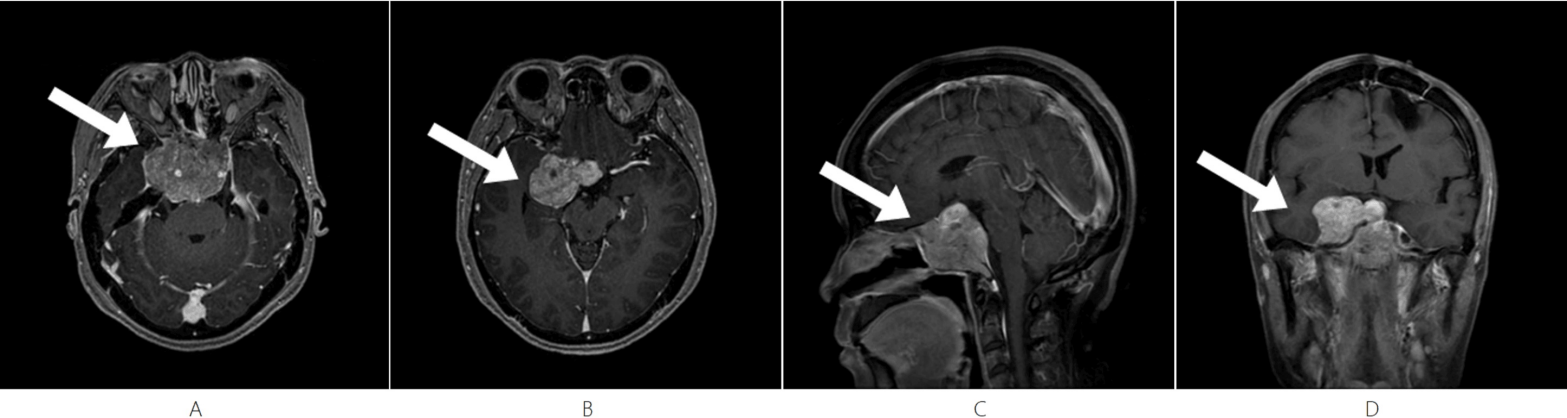
Immediately Post-IMPT - November 2023 The volume of the sellar mass is slightly reduced compared to the pre-treatment scan. (A, B) Axial T1-weighted post-contrast images show a sellar mass with heterogeneous enhancement (arrow). (C) Sagittal T1-weighted post-contrast image shows the lesion with heterogeneous mild-to-moderate enhancement (arrow). (D) Coronal T1-weighted post-contrast image shows the lesion with heterogeneous mild-to-moderate enhancement (arrow). IMPT, intensity-modulated proton therapy

An MRI was performed in November 2023, immediately upon completion of IMPT, to establish a new post-treatment baseline for future comparison. The scan showed the tumor measuring approximately 4.0 cm × 5.2 cm × 3.9 cm (Figure 5). The volume of the sellar mass was noted to be slightly reduced compared to the pre-treatment scan, with persistent heterogeneous mild-to-moderate enhancement and features like the "snowman sign," and continued encasement of critical structures. This scan also noted post-surgical changes from a prior left frontal lobe cavernous hemangioma resection and sinusitis.

**Figure 6:**
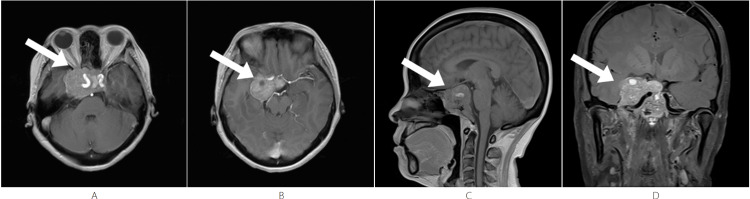
Nine Months Post-IMPT - July 2024 The volume of the sellar mass is slightly reduced compared to the previous scan; the lesion anterior to the left midbrain is smaller; compression of the brainstem and optic chiasm is slightly relieved. (A, B) Axial T1-weighted post-contrast images show that the sellar mass is slightly smaller compared to previous scans; the lesion anterior to the left midbrain is smaller (arrow). (C) Sagittal T1-weighted post-contrast image shows the lesion with heterogeneous mild-to-moderate enhancement (arrow). (D) Coronal T1-weighted post-contrast image shows the lesion with heterogeneous mild-to-moderate enhancement (arrow). IMPT, intensity-modulated proton therapy

Follow-up MRIs were conducted post-IMPT. At nine months post-IMPT (July 2024), the sellar mass showed a slight reduction in volume compared to previous scans, and the lesion anterior to the left midbrain was smaller (Figures 6A, 6B). Heterogeneous mild-to-moderate enhancement persisted (Figures 6C, 6D), and compression of the brainstem and optic chiasm was slightly relieved. 

**Figure 7:**
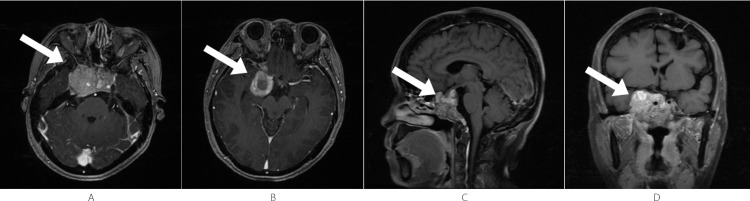
Sixteen Months Post-IMPT - March 2025 The sellar lesion is significantly smaller compared to the previous scan; the lesion anterior to the left midbrain is significantly smaller; compression of the brainstem and optic chiasm is significantly relieved. (A, B) Axial T1-weighted post-contrast images show that the sellar lesion is significantly smaller compared to previous scans; the lesion anterior to the left midbrain is significantly smaller (arrow). (C) Sagittal T1-weighted post-contrast image shows the lesion with heterogeneous mild-to-moderate enhancement (arrow). (D): Coronal T1-weighted post-contrast image shows the lesion with heterogeneous mild-to-moderate enhancement (arrow). IMPT, intensity-modulated proton therapy

At approximately 16 months post-IMPT (March 2025), the sellar lesion was significantly smaller compared to previous scans, and the lesion anterior to the left midbrain was also significantly smaller, measuring approximately 3.3 cm × 5.0 cm × 3.9 cm (Figures 7A, 7B). Compression of the brainstem and optic chiasm was significantly relieved (Figure 7), signifying a positive therapeutic response. This case highlights IMPT's successful application for tumor control in a complex, giant, recurrent NFPA unsuitable for further surgery. A follow-up eye exam prior to discharge showed that her visual acuity and fields remained stable.

## Discussion

The therapeutic management of NFPAs, particularly those characterized as giant (>4 cm) and exhibiting compressive properties or extension into critical areas, presents substantial clinical complexities [8,9]. Although surgical intervention constitutes the primary modality, the attainment of complete resection frequently proves challenging due to involvement of critical anatomical structures, consequently leading to elevated rates of tumor recurrence [11,23]. RT serves as a foundational element in the management of residual, recurrent, or unresectable NFPAs, demonstrating excellent long-term tumor control efficacy [11,24]. Nevertheless, the anatomical juxtaposition of pituitary adenomas to vital structures - including the optic apparatus (which includes the optic nerves, chiasm, and optic tracts), brainstem, temporal lobes, and hypothalamus - mandates the utilization of highly conformal treatment techniques to mitigate the risk of significant neurological, visual, and endocrine toxicities [25,26]. Furthermore, considering the benign histological nature of NFPAs and the potential for prolonged patient survival, the minimization of late-onset adverse effects, such as secondary malignancies and neurocognitive impairment, assumes paramount importance [11,25]. In the specific case presented herein, the documented extension into and compression of the optic chiasm, brainstem, and cavernous sinus established a high-risk clinical scenario wherein the safe and effective delivery of radiation dose via conventional X-ray therapy or SRS was considered particularly problematic [25,26].

Evolution of RT techniques for NFPAs

RT for NFPAs has evolved significantly, aiming to improve the balance between tumor control and toxicity [10,11]. CFRT, using photons, has long served as an adjuvant or definitive treatment for NFPAs [10,24]. While achieving good long-term local control rates (85%-95%), extended follow-up (over 10 years) reveals potential late adverse events, including hypopituitarism, optic neuropathy, and rare secondary malignancies [10,27]. Consideration of these late toxicities is particularly crucial for younger patients or those with anticipated long-term survival, as minimizing iatrogenic harm is paramount [28,29]. SRS delivers a high dose (typically 12-14 Gy for NFPAs) in a single fraction with high precision [27,30]. It is suitable for smaller tumors (<3 cm), located safely away from the optic apparatus (typically >2-5 mm distance), and achieves high local control rates (>90%) [15,30]. However, its proximity to the optic nerves poses a risk of visual impairment, necessitating careful patient selection and dose planning [31,32]. Stereotactic radiotherapy (SRT), similar to SRS in precision, utilizes fractionation (typically 45-54 Gy) to protect critical structures like the optic nerves and brainstem [27,33]. This allows treatment of larger or critically located tumors, achieving high local control (>90%) [26]. For functioning adenomas, SRT can achieve hormone normalization rates of 30%-60%, although this process often takes several years of follow-up [15,26]. Advanced photon techniques like IMRT/VMAT provide high conformity but may increase SMN risk due to a larger low-dose radiation volume ("low-dose bath") compared to simpler methods or PBT [29,34,35].

PBT: physical principles and dosimetric advantages

PBT utilizes protons, whose Bragg peak characteristic deposits maximal energy at a specific depth, with minimal exit dose - unlike photons, which deposit dose continuously [17,19]. This allows superior sparing of normal tissues distal and lateral to the target, crucial for tumors near multiple organs at risk (OARs), as seen in NFPAs [19,28,36]. This targeted energy deposition reduces dose to healthy tissues, lowering long-term side effect risks [28,37]. By reducing the irradiated volume - particularly low/intermediate doses - PBT lowers the integral dose, theoretically decreasing the risk of late effects like SMNs [20,34,37]. An RBE of 1.1 is typically used to equate proton dose (Gy RBE or CGE (Cobalt Gray Equivalent)) to photon dose biologically [19,29,38].

Clinical evidence and comparative effectiveness

The clinical evidence base evaluating PBT for pituitary adenomas is less extensive than that for photon RT, consisting mainly of retrospective, single-center series, often using older passive scattering proton therapy (PSPT) techniques, with a notable absence of direct comparative randomized controlled trials (RCTs) [29,39]. Despite these limitations, existing PBT studies (FSRT and SRS) consistently report excellent long-term tumor control rates (>90%-98%), comparable to modern photon therapies [27-29]. This suggests the primary distinction between modalities lies less in tumor control efficacy and more in the potential for reduced treatment-related toxicity.

Regarding Toxicities

Hypopituitarism remains the most frequent late adverse effect following PBT, with incidence rates similar to photon RT (e.g., up to 62% at five years post-proton stereotactic radiosurgery (PSRS)) [4,28,40]. High baseline endocrine dysfunction rates confound interpretation, and current evidence does not clearly show PBT reduces this risk compared to photons, likely because tumor control doses often exceed pituitary tolerance [4,27,28]. Lifelong endocrine surveillance is essential.

Radiation-induced optic neuropathy (RON) risk is low (<5%) with PBT, similar to modern photon RT, assuming adherence to dose constraints [29,40]. PBT’s potential for lower maximum doses to optic structures might offer an advantage in high-risk cases, though the clinical impact may be limited given the already low rates with photons [28,32,41].

Other toxicities, like radiation necrosis, other cranial neuropathies, and cerebrovascular accident (CVA), are infrequent with modern PBT, comparable to advanced photon therapy [15,17,25].

Neurocognitive effects: PBT/IMPT offers a theoretical advantage due to better sparing of temporal lobes and hippocampi, compared to IMRT/VMAT [29,42]. Some pediatric data suggest potential cognitive benefits, but direct comparative data for pituitary adenomas are lacking [43,44].

Secondary malignant neoplasms (SMNs): PBT/IMPT holds its most significant theoretical advantage here due to reduced integral dose. Models predict substantially lower SMN risk, potentially >50% reduction, compared to photons [29,45]. IMPT may offer further reduction [29]. While clinical validation requires very long-term follow-up (current data insufficient, though some reports are encouraging), this potential benefit strongly supports PBT/IMPT consideration, especially in younger patients [28,29,42].

Rationale for PBT/IMPT in younger patients and complex cases

The prospective reduction in SMN risk serves as a principal justification for considering PBT/IMPT, particularly for younger patients (including individuals in their 40s or 50s, as exemplified by the patient in this report, as well as even younger cohorts), who possess extended life expectancies following treatment for a benign condition [29]. This theoretical reduction in SMN risk assumes heightened clinical significance in relatively younger patients, such as the 49-year-old individual presented, for whom decades of potential life expectancy remain following treatment of a benign condition. Minimizing late iatrogenic mortality attributable to secondary cancers is, therefore, a critical consideration favoring techniques offering lower integral radiation doses [15,25,29]. From the perspective of mitigating secondary cancer risk, PBT - especially IMPT - emerges as a potentially superior alternative compared to conventional X-ray therapy. The potential for reduced long-term side effects, including cognitive decline and visual impairments often associated with traditional RT, further supports the consideration of PBT in younger patients or those with tumors near critical structures [25,29].

Furthermore, in patients presenting with giant tumors, those with compressive effects, or geometrically complex tumors that abut multiple critical OARs (as illustrated in the present case), the superior dose conformity achievable with PBT - and particularly IMPT - confers a significant advantage [21,46]. IMPT provides the technical capacity to deliver the requisite tumoricidal radiation dose while concurrently maximizing the sparing of adjacent radiosensitive structures. This capability may translate into a reduced risk of severe neurological or visual complications, relative to what might be attainable with photon-based techniques, without compromising target coverage [19,29,45]. Studies comparing PBS IMPT to IMRT for skull base tumors have shown superior protection of critical OARs with IMPT [15,38,43]. PBT also represents a valuable therapeutic option in the context of re-irradiation, where cumulative dose constraints imposed on OARs constitute a major limiting factor [29,47].

Management strategy and the role of active surveillance

A critical aspect of this case was the decision to employ active surveillance following the initial surgeries in 2014 and 2017. While adjuvant RT is a well-established treatment for residual or recurrent NFPAs to ensure long-term tumor control [23], the management of asymptomatic post-operative residual tumors requires an individualized approach and remains a matter of debate [48].

In clinical practice, the decision for immediate RT must be weighed against its potential long-term toxicities, including hypopituitarism, optic neuropathy, and the risk of SMNs. Given that NFPAs are typically slow-growing, benign neoplasms, a strategy of active surveillance with close radiological follow-up is considered a viable management option for a select group of patients - particularly those who are younger and clinically stable [5,10,49]. The rationale for this approach is to postpone radiation-induced morbidity until intervention is necessitated by clear evidence of tumor progression. Observational data show that tumor enlargement in untreated residual macroadenomas occurs in approximately 23%-24% of cases over several years, with the majority remaining stable [5,50]. A recent large retrospective study directly comparing management strategies concluded that a “wait-and-see” attitude is a valid option, as adjuvant RT was not superior to salvage RT upon progression. The same study noted that, even among patients with a residual tumor, more than half did not require further treatment at 10 years [48].

In the case of our patient, the decisions for active surveillance were made after multidisciplinary team (MDT) discussions based on this evidence, prioritizing the patient's quality of life and aiming to defer the risks of RT. However, the significant tumor progression observed in 2023 underscored the limitations of this strategy for this particular patient and created a challenging clinical scenario. It was this progression to a giant, unresectable mass compressing multiple critical structures that ultimately highlighted the necessity for a highly conformal radiotherapeutic modality like IMPT, capable of delivering a curative dose while maximally sparing surrounding organs.

Limitations and future directions

The current evidence supporting PBT for pituitary adenomas is primarily limited by retrospective study designs, heterogeneity, lack of direct RCT comparisons with modern photon RT, and insufficient follow-up for assessing late effects like SMNs [28,37]. Specifically, clinical data on IMPT outcomes remain scarce [29]. While dosimetric studies consistently show PBT/IMPT advantages in OAR sparing and reduced integral dose [10,37,38], translating these into proven clinical benefits requires further rigorous investigation. Prospective registries, multi-center collaborations, and comparative effectiveness research are crucial to identify optimal patient subgroups and clarify long-term impacts on toxicities - including endocrine, visual, and neurocognitive function, and SMN incidence - relative to advanced photon therapies. Practical challenges, including higher costs and limited accessibility of PBT, also persist [28]. Long-term follow-up is particularly vital for evaluating outcomes in slowly growing tumors [7,33].

## Conclusions

RT is an effective modality for controlling NFPAs, especially residual, recurrent, or unresectable tumors. PBT offers distinct physical advantages over photons, notably the Bragg peak, which reduces dose to normal tissues and lowers integral dose. IMPT, the most advanced PBT technique, provides superior dose conformity, particularly for complex tumors near critical structures.

This case report demonstrates IMPT’s successful application in a challenging, giant, recurrent, unresectable NFPA involving critical structures, where conventional RT posed significant difficulties. The primary rationale for PBT/IMPT often involves mitigating long-term toxicities, especially the theoretical reduction in SMN risk, making it particularly attractive for younger patients. Potential benefits may also include neurocognitive preservation and reduced optic neuropathy risk. Although hypopituitarism remains common across modalities, IMPT is a valuable and safe strategy for complex NFPAs, optimizing the therapeutic ratio. Future prospective studies are crucial to validate IMPT’s long-term clinical advantages and solidify its role in pituitary adenoma treatment algorithms.

## References

[1] Ntali G, Wass JA (2018). Epidemiology, clinical presentation and diagnosis of non-functioning pituitary adenomas. Pituitary.

[2] Ezzat S, Asa SL, Couldwell WT, Barr CE, Dodge WE, Vance ML, McCutcheon IE (2004). The prevalence of pituitary adenomas: a systematic review. Cancer.

[3] Ferrante E, Ferraroni M, Castrignanò T (2006). Non-functioning pituitary adenoma database: a useful resource to improve the clinical management of pituitary tumors. Eur J Endocrinol.

[4] Mehta GU, Lonser RR (2017). Management of hormone-secreting pituitary adenomas. Neuro Oncol.

[5] Molitch ME (2017). Diagnosis and treatment of pituitary adenomas: a review. JAMA.

[6] Brada M, Ajithkumar TV, Minniti G (2004). Radiosurgery for pituitary adenomas. Clin Endocrinol (Oxf).

[7] Gopalan R, Schlesinger D, Vance ML, Laws E, Sheehan J (2011). Long-term outcomes after gamma knife radiosurgery for patients with a nonfunctioning pituitary adenoma. Neurosurgery.

[8] Ceylan S, Sen HE, Ozsoy B (2022). Endoscopic approach for giant pituitary adenoma: clinical outcomes of 205 patients and comparison of two proposed classification systems for preoperative prediction of extent of resection. J Neurosurg.

[9] Emengen A, Yilmaz E, Gokbel A (2025). Refining endoscopic and combined surgical strategies for giant pituitary adenomas: a tertiary-center evaluation of 49 cases over the past year. Cancers (Basel).

[10] AlMalki MH, Ahmad MM, Brema I (2020). Contemporary management of clinically non-functioning pituitary adenomas: a clinical review. Clin Med Insights Endocrinol Diabetes.

[11] Greenman Y, Stern N (2009). Non-functioning pituitary adenomas. Best Pract Res Clin Endocrinol Metab.

[12] Lee CC, Kano H, Yang HC (2014). Initial gamma knife radiosurgery for nonfunctioning pituitary adenomas. J Neurosurg.

[13] Melesy ARA (2021). Endoscopic endonasal surgery for clinically nonfunctioning pituitary adenomas. Open J Mod Neurosurg.

[14] Yu J, Fu J, Li Y (2024). Hypopituitarism after gamma knife radiosurgery for pituitary adenomas: long-term results from a single-center experience. BMC Cancer.

[15] Minniti G, Scaringi C, Enrici RM (2011). Radiation techniques for acromegaly. Radiat Oncol.

[16] McCord MW, Buatti JM, Fennell EM (1997). Radiotherapy for pituitary adenoma: long-term outcome and sequelae. Int J Radiat Oncol Biol Phys.

[17] Salem PP, Chami P, Daou R (2024). Proton radiation therapy: a systematic review of treatment-related side effects and toxicities. Int J Mol Sci.

[18] Hoskin PJ, Bhattacharya IS (2014). Protons and more: state of the art in radiotherapy. Clin Med (Lond).

[19] Mohan R, Grosshans D (2017). Proton therapy - present and future. Adv Drug Deliv Rev.

[20] Allen AM, Pawlicki T, Dong L (2012). An evidence based review of proton beam therapy: the report of ASTRO's emerging technology committee. Radiother Oncol.

[21] Zhang X, Li Y, Pan X (2010). Intensity-modulated proton therapy reduces the dose to normal tissue compared with intensity-modulated radiation therapy or passive scattering proton therapy and enables individualized radical radiotherapy for extensive stage IIIB non-small-cell lung cancer: a virtual clinical study. Int J Radiat Oncol Biol Phys.

[22] Di Ieva A, Rotondo F, Syro LV, Cusimano MD, Kovacs K (2014). Aggressive pituitary adenomas--diagnosis and emerging treatments. Nat Rev Endocrinol.

[23] Brada M, Rajan B, Traish D, Ashley S, Holmes-Sellors PJ, Nussey S, Uttley D (1993). The long-term efficacy of conservative surgery and radiotherapy in the control of pituitary adenomas. Clin Endocrinol (Oxf).

[24] Snead FE, Amdur RJ, Morris CG, Mendenhall WM (2008). Long-term outcomes of radiotherapy for pituitary adenomas. Int J Radiat Oncol Biol Phys.

[25] Del Monte P, Foppiani L, Ruelle A (2007). Clinically non-functioning pituitary macroadenomas in the elderly. Aging Clin Exp Res.

[26] Minniti G, Osti MF, Niyazi M (2016). Target delineation and optimal radiosurgical dose for pituitary tumors. Radiat Oncol.

[27] Loeffler JS, Shih HA (2011). Radiation therapy in the management of pituitary adenomas. J Clin Endocrinol Metab.

[28] Wattson DA, Tanguturi SK, Spiegel DY (2014). Outcomes of proton therapy for patients with functional pituitary adenomas. Int J Radiat Oncol Biol Phys.

[29] Lesueur P, Calugaru V, Nauraye C (2019). Proton therapy for treatment of intracranial benign tumors in adults: A systematic review. Cancer Treat Rev.

[30] Kotecha R, Sahgal A, Rubens M (2020). Stereotactic radiosurgery for non-functioning pituitary adenomas: meta-analysis and International Stereotactic Radiosurgery Society practice opinion. Neuro Oncol.

[31] Albano L, Losa M, Barzaghi LR (2021). Gamma knife radiosurgery for pituitary tumors: a systematic review and meta-analysis. Cancers (Basel).

[32] Pollock BE, Carpenter PC (2003). Stereotactic radiosurgery as an alternative to fractionated radiotherapy for patients with recurrent or residual nonfunctioning pituitary adenomas. Neurosurgery.

[33] Li X, Li Y, Cao Y, Li P, Liang B, Sun J, Feng E (2017). Safety and efficacy of fractionated stereotactic radiotherapy and stereotactic radiosurgery for treatment of pituitary adenomas: a systematic review and meta-analysis. J Neurol Sci.

[34] Newhauser WD, Durante M (2011). Assessing the risk of second malignancies after modern radiotherapy. Nat Rev Cancer.

[35] St Clair WH, Adams JA, Bues M (2004). Advantage of protons compared to conventional X-ray or IMRT in the treatment of a pediatric patient with medulloblastoma. Int J Radiat Oncol Biol Phys.

[36] Murray FR, Snider JW, Bolsi A (2017). Long-term clinical outcomes of pencil beam scanning proton therapy for benign and non-benign intracranial meningiomas. Int J Radiat Oncol Biol Phys.

[37] Chung CS, Yock TI, Nelson K, Xu Y, Keating NL, Tarbell NJ (2013). Incidence of second malignancies among patients treated with proton versus photon radiation. Int J Radiat Oncol Biol Phys.

[38] Sato H, Mizumoto M, Okumura T (2021). Long-term outcomes of patients with unresectable benign meningioma treated with proton beam therapy. J Radiat Res.

[39] Ronson BB, Schulte RW, Han KP, Loredo LN, Slater JM, Slater JD (2006). Fractionated proton beam irradiation of pituitary adenomas. Int J Radiat Oncol Biol Phys.

[40] Paek SH, Downes MB, Bednarz G, Keane WM, Werner-Wasik M, Curran WJ Jr, Andrews DW (2005). Integration of surgery with fractionated stereotactic radiotherapy for treatment of nonfunctioning pituitary macroadenomas. Int J Radiat Oncol Biol Phys.

[41] Iwata H, Sato K, Tatewaki K, Yokota N, Inoue M, Baba Y, Shibamoto Y (2011). Hypofractionated stereotactic radiotherapy with CyberKnife for nonfunctioning pituitary adenoma: high local control with low toxicity. Neuro Oncol.

[42] Eaton BR, Esiashvili N, Kim S (2016). Clinical outcomes among children with standard-risk medulloblastoma treated with proton and photon radiation therapy: a comparison of disease control and overall survival. Int J Radiat Oncol Biol Phys.

[43] Saito T, Mizumoto M, Oshiro Y (2024). Systematic review and meta-analysis of particle beam therapy versus photon radiotherapy for skull base chordoma: TRP-chordoma 2024. Cancers (Basel).

[44] Lassaletta Á, Morales JS, Valenzuela PL (2023). Neurocognitive outcomes in pediatric brain tumors after treatment with proton versus photon radiation: a systematic review and meta-analysis. World J Pediatr.

[45] Hug EB, Pelak M, Frank SJ, Fossati P (2021). A review of particle therapy for skull base tumors: modern considerations and future directions. Int J Part Ther.

[46] Krcek R, Leiser D, García-Marqueta M, Bolsi A, Weber DC (2023). Long term outcome and quality of life of intracranial meningioma patients treated with pencil beam scanning proton therapy. Cancers (Basel).

[47] Petit JH, Biller BM, Coen JJ (2007). Proton stereotactic radiosurgery in management of persistent acromegaly. Endocr Pract.

[48] Charleux T, Vendrely V, Huchet A (2022). Management after initial surgery of nonfunctioning pituitary adenoma: surveillance, radiotherapy or surgery?. Radiat Oncol.

[49] Esposito D, Olsson DS, Ragnarsson O, Buchfelder M, Skoglund T, Johannsson G (2019). Non-functioning pituitary adenomas: indications for pituitary surgery and post-surgical management. Pituitary.

[50] Huang W, Molitch ME (2018). Management of nonfunctioning pituitary adenomas (NFAs): observation. Pituitary.

